# Hemolysis in Hemodialysis, Secondary to Severe Vena Cava Stenosis

**DOI:** 10.7759/cureus.15156

**Published:** 2021-05-21

**Authors:** Dahyana Cadavid Aljure, Sergio Alvarez-Vallejo, Gloria Posada-Alvarez, Eliana Ruiz-Aguilar, Lina Higuita-Urrego, Catalina Guerra-Alvarez, Sandra Marin-Durango, Catalina Ocampo-Kohn, John Fredy Nieto-Rios, Arbey Aristizabal-Alzate, Gustavo Zuluaga-Valencia

**Affiliations:** 1 Nephrology and Kidney Transplant Department, Hospital Pablo Tobón Uribe, Medellín, COL; 2 Interventional Radiology Department, Hospital Pablo Tobón Uribe, Medellín, COL; 3 Dialysis Nursing Department, Hospital Pablo Tobón Uribe, Medellín, COL; 4 Nephrology and Kidney Transplant Department, Nephrology Section, Department of Internal Medicine, Hospital Pablo Tobón Uribe, University of Antioquia, Medellín, COL

**Keywords:** hemodialysis access, hemolysis, stenosis, anemia, blood circulation, complications

## Abstract

Complications in hemodialysis patients are increasingly rare thanks to advances in technology, including more compatible membranes, more flexible lines, safety in water treatments, alarms in the circuit, and standardization in dialysate fluids plus exhaustive chemical and microbiological tests. In addition, it is highly unusual having hemolysis on hemodialysis; however, it is a life-threatening complication, so the cause must be identified and early managed. The etiology can be chemical or mechanical; however, so far, there are no reports in the literature of an association with severe stenosis of the vena cava, as it is described in the case reported here, where a patient presented hemolysis in two hemodialysis sessions, without initially being possible to find the cause; the only identifiable factor was that he had a dysfunctional tunneled jugular catheter, with a history of difficult vascular access. The patient underwent interventional radiology, finding 99% stenosis of the vena cava, which prevented the passage of the contrast agent to the atrium. Angioplasty and catheter replacement were performed, with a resolution of the complication; the subsequent dialysis therapies were satisfactory.

## Introduction

According to the High-Cost Account in 2019 in Colombia, there was a prevalence of 925,996 people diagnosed with chronic kidney disease in any of its stages, of which 43,153 required dialysis, with a prevalence of 86.12 cases per 100,000 inhabitants [1]; these patients have a high predisposition to complications, morbidity, and mortality.

Hemolysis in hemodialysis is an infrequent complication [2], but potentially fatal, which implies making imperative early recognition and active search for the etiology, which can be chemical or mechanical; the latter being more related to the extracorporeal circuit such as mispositioned or kinked lines or hemolysis due to needles [2-4]; however, there is no report of hemolysis secondary to stenosis of the vena cava. Next, we describe the first case reported for this cause.

## Case presentation

An 80-year-old man presented with a history of arterial hypertension, chronic obstructive pulmonary disease, type 2 diabetes mellitus, revascularized ischemic heart disease, a cardioverter-defibrillator carrier implanted five years ago, and category G5D diabetic kidney disease. He was on hemodialysis through a right tunneled jugular catheter inserted two years ago and had a history of very difficult vascular access. He was hospitalized for a seizure syndrome associated with focal epilepsy. During the hospitalization, he continued the hemodialysis program three times a week with sustained low-efficiency dialysis (SLED) therapy with a Genius machine (Genius®, Fresenius Medical Care, Bad Homburg, Germany); the first hospital dialysis was difficult and hard to perform due to dysfunctional vascular access, although it was achieved with inversion of the lines (blood recovery through the distal lumen of the catheter, that is, venous port and return through the proximal arterial lumen). During the second dialysis, an hour and a half after starting the therapy, hematic dialysis fluid and ultrafiltrate were observed so the therapy was quickly suspended, avoiding the return of blood to the patient. Paraclinical tests were taken, finding a decrease in hemoglobin, an increase in both potassium and lactate dehydrogenase (Table 1).

**Table 1:** Evolution of laboratory analyses LDH: lactate dehydrogenase, INR: international normalized ratio

Variable	Upon Admission	1st Event Of Hemolysis	2^nd^ Event Of Hemolysis	Upon Discharge	Reference Values
Hemoglobin	9.6	8.8	7.5	8.6	13-17 g/dl
Hematocrit	30.4%	27%	23%	26%	40-50%
Platelets	406.000	376.000	278.000	272.000	150-450.000 mm^3^
INR/prothrombin time	*	1.09/12.4	1.18/13.4	*	0.9-1.2/9.7-13 seconds
Partial thromboplastin time	*	31.1	73.3	*	26-36.9 seconds
Total bilirubin	0.53	0.7	*	*	0.2-1.2 mg/dl
Direct bilirubin	0.13	0.22	*	*	0.1-0.5 mg/dl
LDH	218	837	715	560	125-243 U/l
Potassium	5.7	6.35	5.8	3.99	3.5-5.0 mmol/l
Blood urea nitrogen	53	87	62	48	8.4-25.7 mg/dl

**Figure 1:**
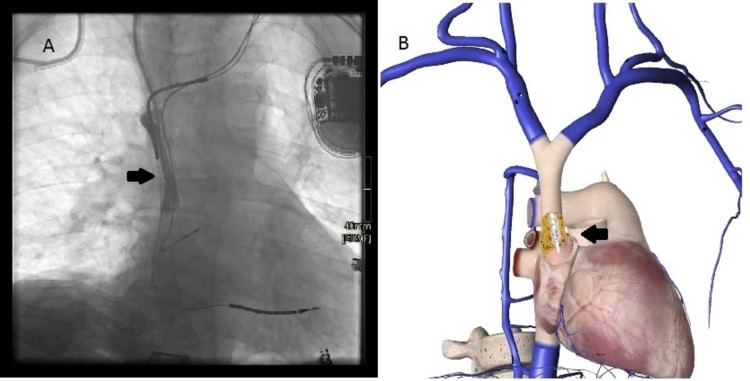
Vena cava atenosis A. Cavography performed from the proximal line of the catheter demonstrates stenosis of the superior vena cava without passage of the contrast medium to the atrium, as well as recirculation of the contrast agent in a 6-cm dead space in the vena cava. B. Schematic representation of the recirculation phenomenon and possible friction secondary to stenosis of the superior vena cava. 1B: Self-created by the authors and edited using the application "The essential anatomy 5, version number: 5.0.8."

The connection lines, temperature, and dialysis liquid were then evaluated with physicochemical tests of the water (chlorine, copper, nitrates, etc.), finding the hemodialysis equipment in an optimal state, without contaminants in the water. In addition, there were no similar events in other hospital patients and there were no predisposing conditions in the patient such as medications, active infection, or diagnosed diseases associated with hemolysis. For these reasons, he was reconnected to renal support therapy two days later, repeatedly presenting hematic dialysis fluid. Since the etiology of the hemolysis was not initially found, and the only problem at that moment was a dysfunctional catheter, a review of the access was requested by interventional radiology. It was found that the proximal line was trapped in a 6-cm-long space in the superior vena cava, inside severe stenosis of 99% of the veno-atrial junction, without free passage of contrast agent, with the flow circulating in a dead space; however, the distal pathway was patent in the right atrium (Figures 1A-1B).

**Figure 2:**
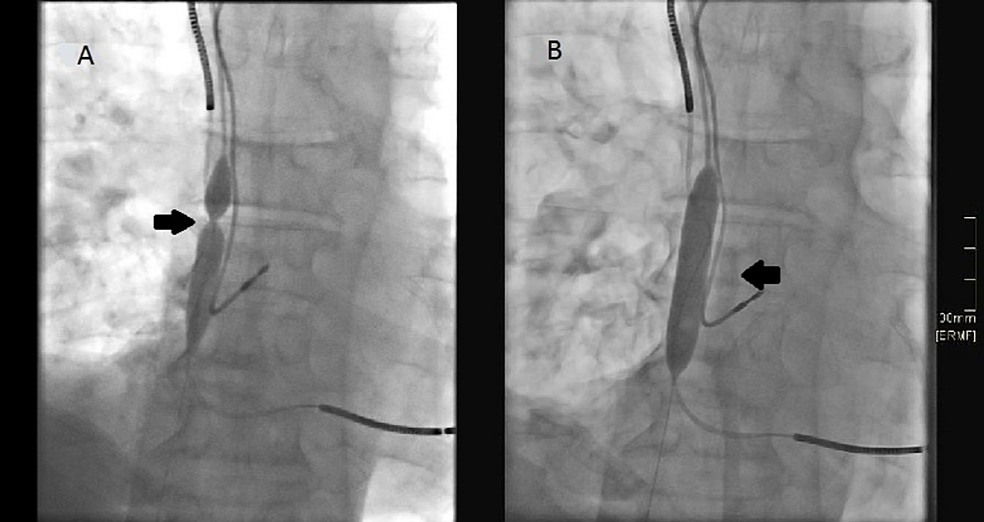
Angioplasty of superior vena cava A. Angioplasty was performed with a 10x40 mm balloon. B. The fibrosis that caused the stenosis was broken, restoring flow through the superior vena cava.

It was decided to perform angioplasty of the vena cava. A 10x40 mm balloon (0.035” Balloon Dilatation Catheter, Boston Scientific, Marlborough, Massachusetts) was used (Figure 2A), demonstrating the disappearance of the waist in the stenosis (Figure 2B). A new tunnel was made, and a new 14frx28 cm catheter (Medcomp®️ Split Cath®️, Medical Components, Inc., Harleysville, Pennsylvania) was implanted in the right jugular vein, with satisfactory flows obtained in both routes (Figures 2A-2B); with this, the following dialyses were uncomplicated and there were no more hemolysis events.

## Discussion

Hemolysis in hemodialysis, although infrequent, is an important cause of morbidity and mortality due to the complications that it can produce, such as profound anemia, arrhythmias, acute coronary syndrome, respiratory distress syndrome, and severe necrotizing pancreatitis, among others, which implies performing the prompt recognition, analysis of etiology, and correction of predisposing factors. Hemolysis can be profound, requires transfusion support, produces hemodynamic instability, and may even require advanced support [5].

In severe acute or subacute hemolysis, the presenting symptoms may vary, the most common being: anorexia, nausea, emesis, abdominal pain, headache, lethargy, chills, diaphoresis, hypotension or hypertension, dyspnea, angina, and hematuria [2]. Depending on the cause of hemolysis, these symptoms may occur within the initial 30 minutes of dialysis, or they may have a prolonged course. The onset of clinical manifestations also varies according to the underlying etiology of hemolysis [2]. Once hemolysis has been detected, it is recommended to stop dialysis immediately, clamping the bloodlines, not returning the blood in order to avoid increasing the risk of hyperkalemia, treating complications, as well as investigating the source [3,5].

The causes of hemolysis described so far can be divided into: chemical agents, such as contamination of the dialysis fluid by chlorine, copper, zinc, chloramines, and formaldehyde; patient-specific causes, such as malignant hypertension, autoimmunity, sickle cell anemia, glucose-6-phosphate dehydrogenase deficiency; or infectious ones like malaria; some medications, such as aspirin, penicillins, cephalosporins, sulfonamides, sulfones, nitrofurantoin, primaquine, quinidine, hydralazine, certain derivatives of vitamin K [2]; and finally, mechanical causes, such as fibers on the surface of old dialysis membranes, overheating of the dialysate fluid, error in the manufacture of the circuit, kinking or bad position of the lines, stress due to low-caliber needles, increase in negative pre-pump pressure [6], small diameter of the hoses [5,7], and there are some anecdotal reports of the use of subclavian catheters associated with partial occlusion of the catheter due to thrombi or clots at the tip of the catheter [8].

So far, there are no reports of hemolysis in hemodialysis due to mechanical causes secondary to vena cava stenosis, as described in this clinical case. The pathophysiological theory that we assume explains hemolysis in relation to stenosis of 99% of the superior vena cava, which prevented the free passage of blood to the atrium, generating a dead space where the proximal line of the catheter (arterial lumen) was trapped, with the fragmentation of red blood cells in this area, which could be aggravated by the increase in pre-pump pressure due to the difficulty of flow; in addition, it could also be increased for having to invert the ports for the connection in the first dialysis (Figures 1A-1B.). When dialysis was carried out on a Genius machine, the return of hematic dialysis fluid and ultrafiltrate could be seen in the glass container. Despite an exhaustive search for the different causes of hemolysis, no alterations were found, and there were no more reported cases of hemolysis in the hospital (where an approximate average of 15 daily dialyses are performed in hospitalized patients). Due to all of the above, and given the particular problem that the patient presented, which was the dysfunctionality of the vascular access, knowing that one of the consequences of the use of long-term dialysis catheters is venous stenosis and thrombosis [9] and that, additionally, this patient was a carrier of a cardioverter-defibrillator, which could be contributing to vascular complications [10], a revision of the catheter was required, finding the described stenosis. After vena cava angioplasty and catheter change (Figures 2A-2B). dialysis could be carried out without problems, and there were no new episodes of hemolysis; therefore, the cause was resolved. An active search of the etiology for an early and appropriate intervention reduces the risk of morbidity and mortality, which is already high in patients with kidney disease on dialysis [5,7,11].

## Conclusions

Despite the fact that complications in hemodialysis are becoming less frequent, it is important to recognize them and actively look for them. Hemolysis in hemodialysis is potentially fatal and must be recognized. A broad differential diagnosis should be made in search of the etiology such as chemical causes, mechanics, or pathology specific to the patient. It must be managed properly, with no interruption in therapy, avoiding blood return from the circuit to the patient, performing an adequate hemodynamic and cardiovascular evaluation, correcting secondary hyperkalemia, and defining whether a blood transfusion is necessary. The cornerstone is to identify and correct the cause, to avoid events that lead to increased morbidity and mortality in hemodialysis patients.
